# Supplementary material for: Genomic locus modulating corneal thickness in the mouse identifies POU6F2 as a potential risk of developing glaucoma
Source: PLoS Genet. 2018 Jan 25;14(1):e1007145. doi: 10.1371/journal.pgen.1007145 (PMC5784889; doi:10.1371/journal.pgen.1007145)
Supplement: S1 Data — We examined the cornea and the eye cup for the expression of the transcripts monitored by three of the probes designated as cis-QTLs in the whole eye dataset. The expression level of Mplkip and Cdk13 is considerably higher (3- to 5-fold) in the eye cup relative to the cornea per ng of cDNA. We next measured the RNA content of the cornea in six independent biological samples relative to the eye cup in four independent biological samples. The cornea contained approximately 1.92% of the total RNA in the eye. To determine the contribution of the RNA expression in the cornea to the signal monitored by microarrays in the whole eye dataset, we multiplied the signal by the relative amount of RNA. The total contribution of corneal signal for Mplkip and Cdk13 to that monitored in the dataset is less than 1%. Thus, if the cis-QTL is real, then it does not represent a corneal QTL for there just is not enough expression in the cornea to be monitored in a whole eye sample (Figure A). (DOCX) [file pgen.1007145.s001.docx]

**S1_Data, Appendix**

Expression of *Mplkip* and *Cdk13* in the Cornea and the Eye in the mouse.

We examined the cornea and the eye cup for the expression of the transcripts monitored by three of the probes designated as cis-QTLs whole eye dataset. The expression level of *Mplkip* and *Cdk13* is considerably higher (3- to 5-fold) in the eye cup relative to the cornea per ng of cDNA. We next measured the RNA content of the cornea in six independent biological samples relative to the eye cup in four independent biological samples. The cornea contained approximately 1.92% of the total RNA in the eye. To determine the contribution of the RNA expression in the cornea to the signal monitored microarrays in the whole eye dataset, we multiplied the signal by the relative amount of RNA. The total contribution of corneal signal for *Mplkip* and *Cdk13* to that monitored in the dataset is less than 1%. Thus, if the cis-QTL is real, then it does not represent a corneal QTL for there just is not enough expression in the cornea to be monitored in a whole eye sample (Figure A).


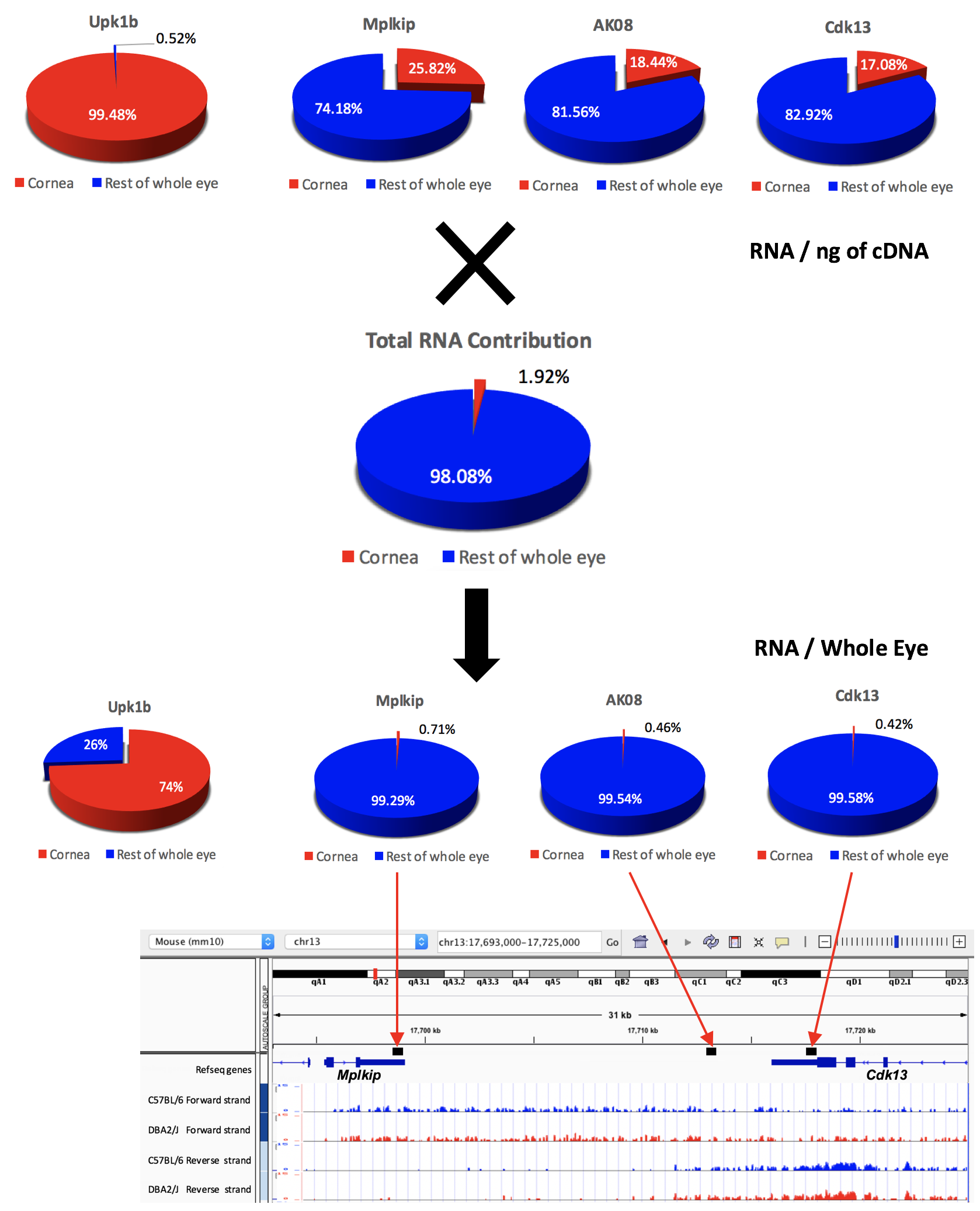


**Figure A.** We conducted a series of RNA isolations and quantitative PCR to determine the relative expression of *Mplkip* and *Cdk13* in the cornea and the eye. Based on ng of cDNA from the tissues, the expression within the cornea was less than 25% of the that in the eye. Almost no expression of the corneal marker *Upk1b* was found in the retina. The total amount of RNA from the cornea in a sample from the eye was only 1.92%. If we determine the relative contribution of corneal *Mplkip* and *Cdk13* to the signal coming from whole eye samples it is less than 1% and thus negligible. The corneal expression of *Mplkip* and *Cdk13* would not be reflected in the whole eye sample. For genes expressed at high levels in the cornea, this is not the case, as it can be seen that the majority of the signal from *Upk1b* in the whole eye sample originates form the cornea.
