# Supplementary material for: Genomic locus modulating corneal thickness in the mouse identifies POU6F2 as a potential risk of developing glaucoma
Source: PLoS Genet. 2018 Jan 25;14(1):e1007145. doi: 10.1371/journal.pgen.1007145 (PMC5784889; doi:10.1371/journal.pgen.1007145)
Supplement: S2 Data — To validate the specificity of rabbit antiserum directed against POU6F2 (MyBiosource, Cat. # MBS9402684) we used in the present study, we first ran an immunoblot and found one major band in the retina sample at approximately 73 kDa, the appropriate size for POU6F2 (lane A). There were no significant bands observed in blots of similar tissue that were stained with secondary antibody only (lane B). Next, we examined four different tissues by PCR, including: retina, brain, colon and salivary gland. We found significant levels of Pou6f2 message in retina and brain, with virtually no Pou6F2 mRNA detected in the other samples. When we examined protein samples from these tissues using immunoblot methods, we observed a 74 kDa band in retina and brain and no bands in colon or salivary gland. This confirmed that the antibody was specific for POU6F2. (DOCX) [file pgen.1007145.s002.docx]

S2_Data , Appendix

To validate the specificity of rabbit antiserum directed against POU6F2 (MyBiosource, Cat. # MBS9402684) we used in the present study, we first ran an immunoblot and found one major band in the retina sample at approximately 73 kDa, the appropriate size for POU6F2 (lane A). There were no significant bands observed in blots of similar tissue that were stained with secondary antibody only (lane B). Next, we examined four different tissues by PCR, including: retina, brain, colon and salivary gland. We found significant levels of *Pou6f2* message in retina and brain, with virtually no *Pou6F2* mRNA detected in the other samples. When we examined protein samples from these tissues using immunoblot methods, we observed a 74 kDa band in retina and brain and no bands in colon or salivary gland. This confirmed that the antibody was specific for POU6F2.


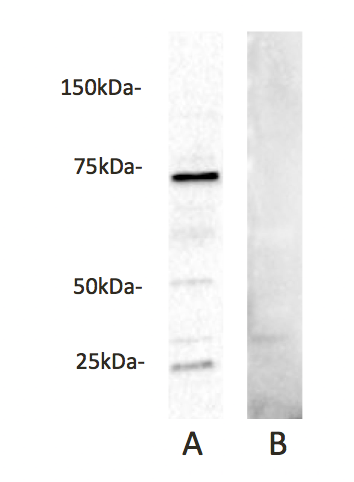


Immunoblots of retina stained with POU6F4 (A) and with secondary antibody only (B). The molecular weights are shown to the left in kDa.
