# Supplementary figures and images for: Genomic locus modulating corneal thickness in the mouse identifies POU6F2 as a potential risk of developing glaucoma
Source: PLoS Genet. 2018 Jan 25;14(1):e1007145. doi: 10.1371/journal.pgen.1007145 (PMC5784889; doi:10.1371/journal.pgen.1007145)

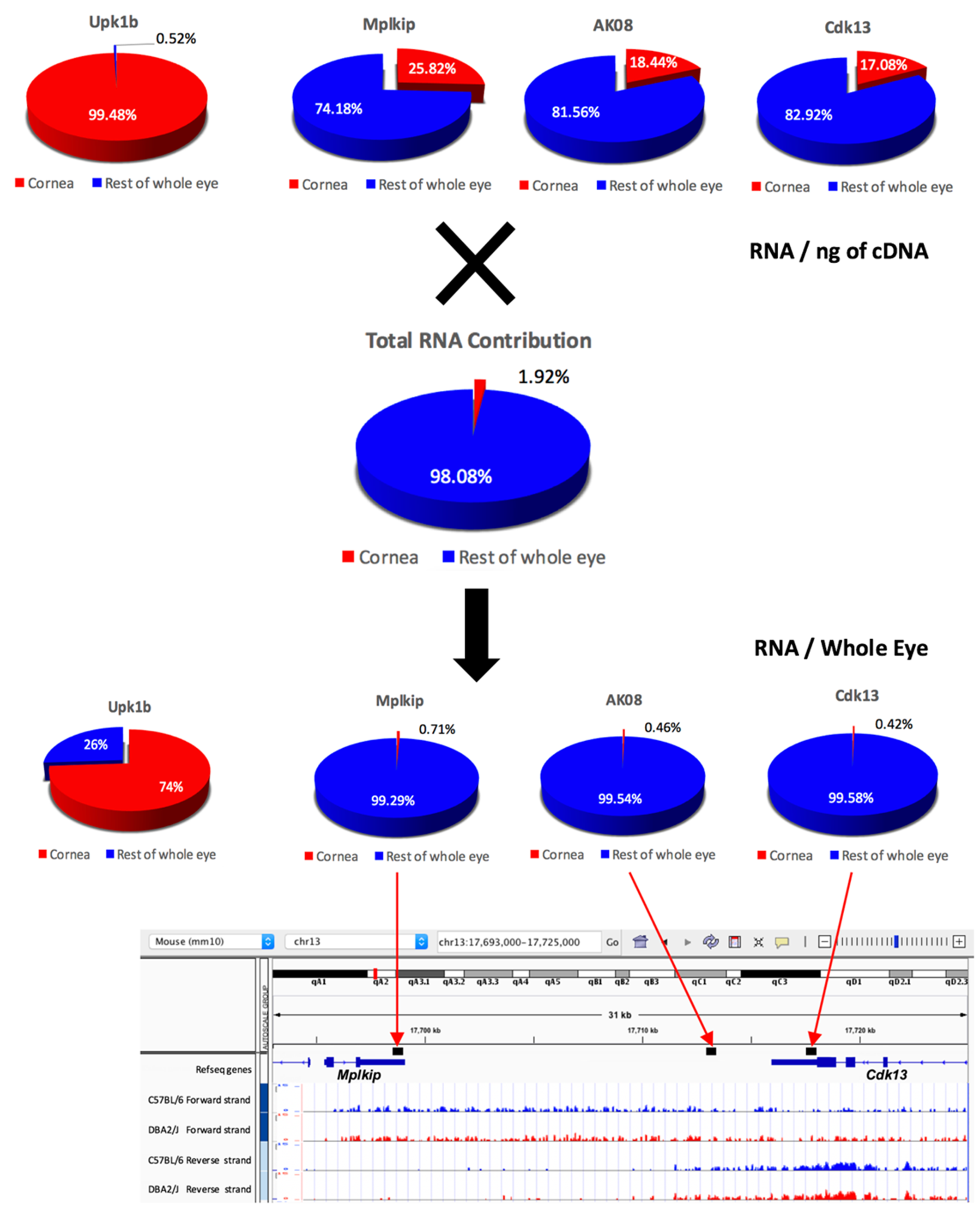

Supplement: S1 Fig — Based on ng of cDNA from the tissues, the expression within the cornea was less than 25% of the that in the eye. Almost no expression of the corneal marker Upk1b was found in the retina. The total amount of RNA from the cornea in a sample from the eye was only 1.92%. If we determine the relative contribution of corneal Mplkip and Cdk13 to the signal coming from whole eye samples it is less than 1% and thus negligible. The corneal expression of Mplkip and Cdk13 would not be reflected in the whole eye sample. For genes expressed at high levels in the cornea, this is not the case, as it can be seen that the majority of the signal from Upk1b in the whole eye sample originates form the cornea. (TIF) [file pgen.1007145.s003.tif]

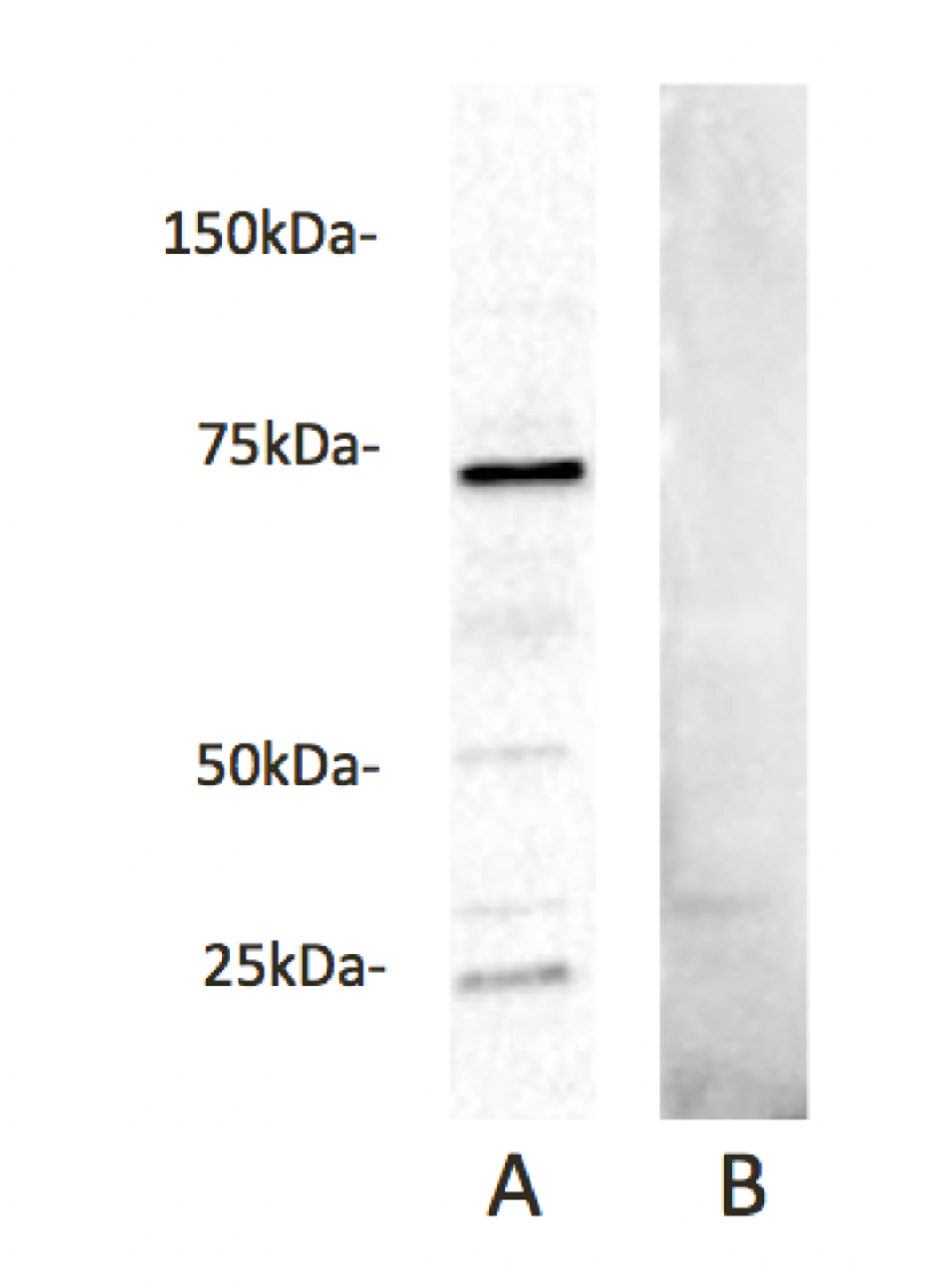

Supplement: S2 Fig — Immunoblots of retina stained with POU6F2 (A) and with secondary antibody only (B). The molecular weights are shown to the left in kDa. (TIF) [file pgen.1007145.s004.tif]

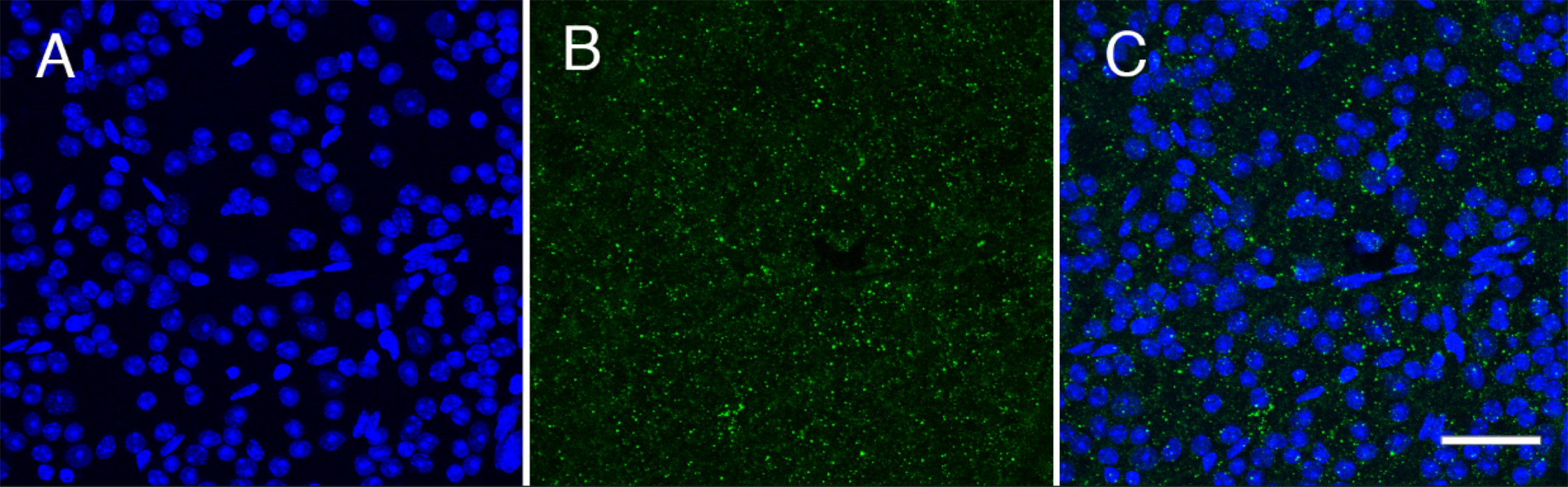

Supplement: S3 Fig — Flat mounts of the retina from mice 28 days after optic nerve crush were stained for POU6F2 (B) and counterstained for TOPRO-3 (A). The two images are merged and presented in C. Notice there is no nuclear labeling in the ganglion cell layer (B and C) and that there is a decreased number of nuclei following optic nerve crush (A and C). All photomicrographs are taken at the same magnification and the scale bar in C represents 50 μm. (TIF) [file pgen.1007145.s005.tif]

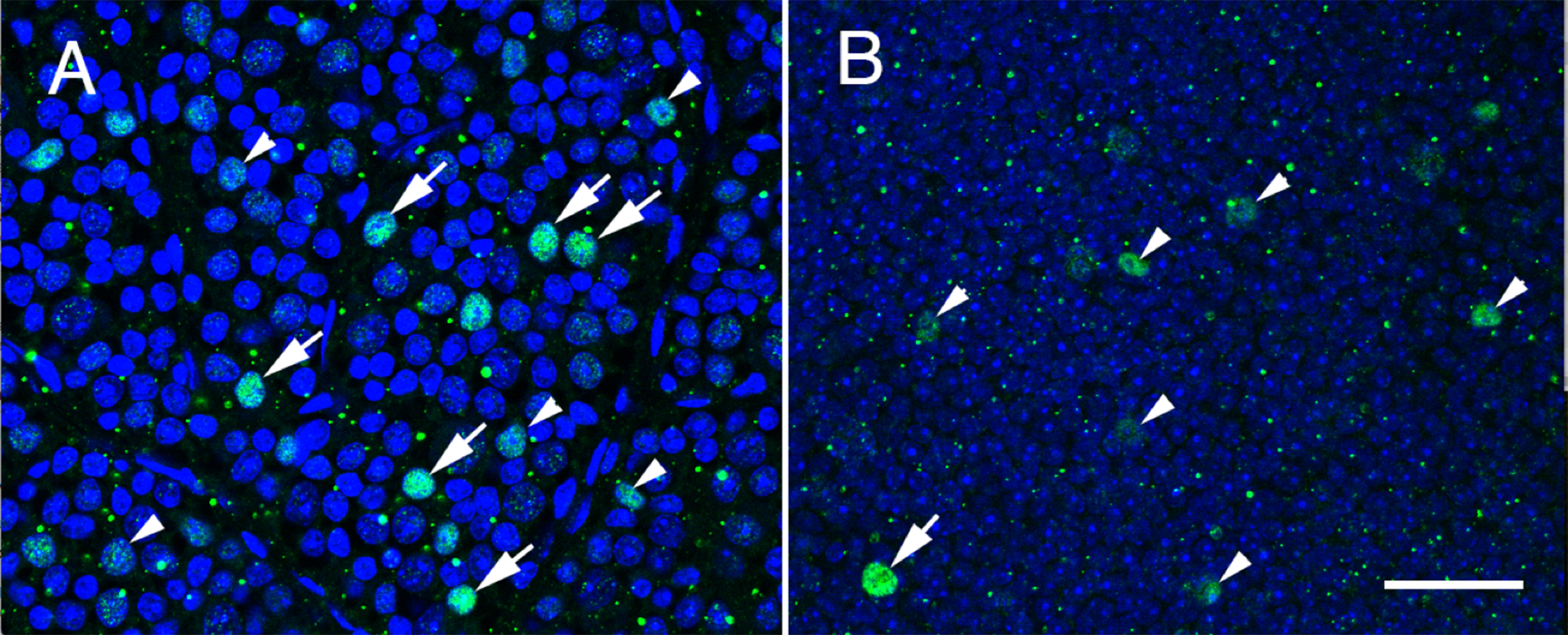

Supplement: S4 Fig — The ganglion cell layer is shown in A and the top of the inner nuclear layer (amacrine cell layer) is shown in B. Notice that brightly labeled cells are observed in the ganglion cell layer and the amacrine cell layer (Arrows). There are also faintly labeled cells in both layers (arrow heads). These data strongly suggest that POU6F2 labels both ganglion cells and amacrine cells. Both A and B are taken at the same magnification and the scale bare in B represents 50 μm. (TIF) [file pgen.1007145.s006.tif]
